# Nanostructures for peroxidases

**DOI:** 10.3389/fmolb.2015.00050

**Published:** 2015-09-03

**Authors:** Ana M. Carmona-Ribeiro, Tatiana Prieto, Iseli L. Nantes

**Affiliations:** ^1^Biocolloids Laboratory, Departamento de Bioquímica, Instituto de Química, Universidade de São PauloSão Paulo, Brazil; ^2^NanoBioMav, Centro de Ciências Naturais e Humanas, Universidade Federal do ABCSanto André, Brazil

**Keywords:** antioxidants, antioxidant enzymes, improved and reusable peroxidase activity, self-assembly, particles, nanotubes, micelles

## Abstract

Peroxidases are enzymes catalyzing redox reactions that cleave peroxides. Their active redox centers have heme, cysteine thiols, selenium, manganese, and other chemical moieties. Peroxidases and their mimetic systems have several technological and biomedical applications such as environment protection, energy production, bioremediation, sensors and immunoassays design, and drug delivery devices. The combination of peroxidases or systems with peroxidase-like activity with nanostructures such as nanoparticles, nanotubes, thin films, liposomes, micelles, nanoflowers, nanorods and others is often an efficient strategy to improve catalytic activity, targeting, and reusability.

## The oxygen paradox in metabolism and the role of peroxidases

The majority of complex organisms on Earth require oxygen for their existence. The use of oxygen in metabolism allows a high-energy output accompanied of the deleterious oxygen effects due to oxygen partial reduction (Halliwell and Gutteridge, 2007). The deleterious effects of oxygen include the oxidative damage to essential biomolecules in the cell such as DNA, proteins and lipids (Sies, 1997; Vertuani et al., 2004). Oxidants are normal products of the aerobic metabolism that reduces molecular oxygen to water. The escape of electrons from the respiratory chain produces the superoxide anion radical, which can form hydrogen peroxide. Hydrogen peroxide can yield the hydroxyl radical, the most reactive pro-oxidant species (Augusto and Miyamoto, 2011). Organic peroxides also derive from the oxidation of lipids and proteins (Sies, 1997). The reactive oxygen species (ROS) are peroxides and free radicals derived from oxygen that are highly reactive toward biomolecules. Free radicals are any atom or molecule that contains unpaired electrons. Hence, as part of the defense against the ROS, organisms developed an intricate network of antioxidants that inhibits the oxidation of other molecules by terminating the reactions leading to the production of free radicals. Antioxidants can remove the free radicals intermediates by oxidizing themselves so that they are often reducing agents such as poly-phenols, tocopherols, carotenes, vitamin A, ubiquinols, thiols, ascorbic acid, and others (Sies, 1997). The antioxidants act in concert with the enzymatic antioxidant defense represented by superoxide dismutases, catalases, glutathione peroxidases, and others (Sies, 1997). An imbalance between oxidants and antioxidants in favor of the oxidants is termed “oxidative stress.” Oxidative stress has been related to the development of several diseases such as Alzheimer's disease (Christen, 2000; Nunomura et al., 2006), diabetes (Giugliano et al., 1996; Davi et al., 2005), rheumatoid arthritis (Hitchon and El-Gabalawy, 2004), Parkinson's disease (Wood-Kaczmar et al., 2006), and neuronal degeneration (Cookson and Shaw, 1999). For example, low density-lipoprotein (LDL) oxidation triggers atherosclerosis leading to cardiovascular disease (Aviram, 2000; Van Gaal et al., 2006). Oxidative damage in DNA can cause cancer (Maynard et al., 2009; Khan et al., 2010). The dietary intake of vitamins E and C, and β-carotene can lower the risk of Alzheimer's disease (Li et al., 2012). However, ROS have also useful cellular functions such as redox signaling (Collins et al., 2012; Reczek and Chandel, 2014; Sies, 2014) or defense against invasive pathogens (Delattin et al., 2014). Hydrogen peroxide (produced from reactions catalyzed by the NADPH oxidases or complex III of the mitochondrial respiratory chain) is a messenger under fine control of peroxiredoxins, glutathione peroxidases and catalase signaling major processes such as cell proliferation, tissue repair, differentiation, inflammation, and aging (Sies, 2014). The main ROS involved in redox signaling is hydrogen peroxide; however, other forms of ROS also contribute as the nitric oxide (NO) generated by NO synthases, which diffuses into mitochondria and modulates mitochondrial function by competing with O_2_ at respiratory complex IV thereby slowing respiration (Collins et al., 2012). ROS are not only a toxic by-product of mitochondrial respiration but also play a role in cellular signaling as for example the role of H_2_O_2_ in thiol oxidation modulating the function of proteins (Collins et al., 2012; Reczek and Chandel, 2014). However, the mechanism by which the ROS signal reaches its target protein in the face of highly reactive and abundant antioxidants in the cell is not fully understood (Reczek and Chandel, 2014). The function of antioxidant systems is not the complete removal of oxidants, but instead the optimization of their intracellular concentrations (Rhee, 2006). Against fungus, azoles, echinocandins and liposomal amphotericin B, besides their specific mode of action, also induce ROS in planktonic and biofilm cells (Delattin et al., 2014). The three main classes of bactericidal antibiotics, namely, the quinolones, the beta-lactams, and the aminoglycosides also induce ROS (Delattin et al., 2014).

The protection available for cells under oxidative stress comes from natural antioxidants and several antioxidant enzymes (Davies, 1995; Sies, 1997). Superoxide released by oxidative processes yields firstly the hydrogen peroxide in a step catalyzed by superoxide dismutase. Then, the second step is the reduction of hydrogen peroxide by catalases and other peroxidases. Since these enzymes play in concert, it is often difficult to ascertain their individual role in the antioxidant defense. The generation of transgenic mice deficient in just one antioxidant enzyme has been a major approach to get further insight on the individual role of each peroxidase (Ho et al., 1998).

**Figure 1:**
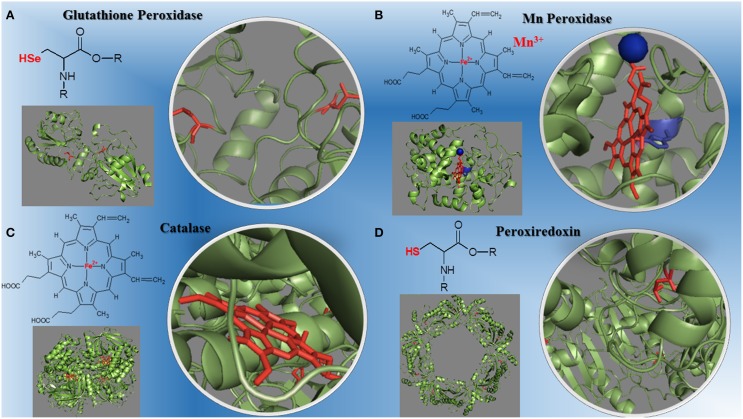
**Structural diversity of peroxidases and their redox centers**. **(A)** The glutathione peroxidase (1GP1-SeH) monomer and its selenium cysteine SeCys-35 (Epp et al., 1983). **(B)** The manganese peroxidase (1YYD) monomer from *Phanerochaete chrysosporium* and its heme iron plus Mn^3+^ (Sundaramoorthy et al., 2005). **(C)** The dimeric catalase (1DGB) structure from human erythrocytes and its heme iron (Putnam et al., 2000). **(D)** The decameric peroxiredoxin (1QMV) from human erythrocytes and its cysteine thiol residues (Schröder et al., 2000). The PDB ID for each protein is inbetween parentheses.

The peroxidases catalyze redox reactions involving peroxides cleavage (Mhamdi et al., 2010; Al Ghouleh et al., 2011; Hall et al., 2011; Nantes et al., 2011; Mishra and Imlay, 2012; Rhee et al., 2012; Shao, 2012; Whittaker, 2012; Zámockı et al., 2012; Rodríguez-Rodríguez et al., 2014). The molecular evolution of peroxidases required for efficient removal of peroxides led to three metallo-enzyme families that differ in oligomers organization, monomer architecture, active site geometry, and catalytic residues. They are: (1) the highly conserved structures of mono-functional heme catalases found in all domains of life; (2) the bi-functional catalase-peroxidases, members of a protein family predominantly present among eubacteria and archaea with only two evolutionary branches found in eukaryotic organisms; (3) the non-heme manganese catalases, a small protein family with old roots, only present in bacteria and archaea (Zámockı et al., 2012). These non-heme manganese catalases represent an environmentally important alternative to heme-containing catalases in antioxidant defense. Manganese catalases contain binuclear manganese complexes in their catalytic site rather than a heme, and cycle between Mn(2)(II,II) and Mn(2)(III,III) states during turnover (Whittaker, 2012). Plants contain several types of H_2_O_2_ –metabolizing proteins; catalases are highly active and do not require cellular reductants as they primarily catalyze a dismutase reaction (Mhamdi et al., 2010). In mammalian cells, six peroxiredoxins isoforms expressed and localized to various cellular compartments function as a peroxidase and contain an active site cysteine that can be oxidized by H_2_O_2_ (Rhee et al., 2012). The peroxiredoxins use a conserved Cys residue to reduce peroxides and are highly expressed in organisms from all kingdoms with 72 structures already determined covering much of the diversity of the family (Hall et al., 2011). Other peroxidases play major roles in cardiovascular, lung and brain diseases (Al Ghouleh et al., 2011; Shao, 2012; Rodríguez-Rodríguez et al., 2014) such as myeloperoxidase, a heme enzyme secreted by human artery wall macrophages which oxidizes apolipoprotein A-I (apoA-I), the major HDL protein, diminishing apoA-I ability to promote cellular cholesterol efflux. The oxidation of specific tyrosine and methionine residues in apoA-I contributes to its loss of activity (Shao, 2012). Peroxiredoxin 6 (Prdx6) is the only mammalian 1-Cys member of the Prdx family. This peroxidase uses the antioxidant tripeptide glutathione (GSH) instead of thioredoxin as the physiological reductant to reduce the oxidized sn-2 fatty acyl group of phospholipids (peroxidase activity) or to hydrolyze the sn-2 ester (alkyl) bond of phospholipids (phospholipase A(2) [PLA(2)] activity). This bi-functional protein has separate active sites for peroxidase and phospholipase PLA activities. These activities are dependent on binding of the protein to phospholipids at acidic pH and to oxidized phospholipids at cytosolic pH (Fisher, 2011). The structures of different peroxidases and their redox centers is on Figure 1.

## Applications for peroxidases and their assemblies

Horseradish peroxidase (HRP) is a much studied and versatile peroxidase with about 600 articles presently available on its assemblies. HRP certainly is the analyst's friend (Ryan et al., 1994; Krainer and Glieder, 2015). This oxidoredutase accepts a wide variety of hydrogen donors to reduce H_2_O_2_, a property applicable to a range of colorimetric, fluorimetric, chemiluminescent, and electrochemical and immuno assays based on HRP activity. However, HRP isolation and purification gives low yields and recombinant HRP obtained in the yeast *Pichia pastoris* is often hyper-glycosylated though an unglycosylated, active and stable HRP variant is now available from site-directed mutagenesis (Capone et al., 2014). The many difficulties inherent to HRP production and purification (Spadiut and Herwig, 2013) essentially emphasize the importance of its functional immobilization in nanostructures for improving activity and allowing reusability.

**Figure 2:**
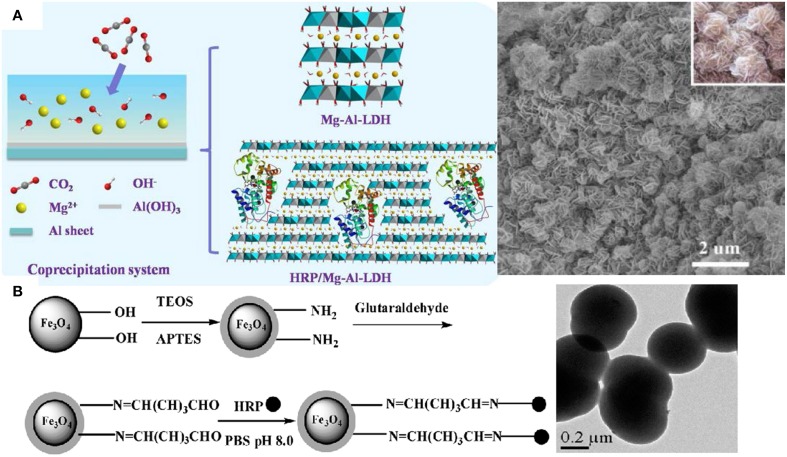
**Nanostructures for immobilizing and reusing peroxidases**. **(A)** Entrapment of HRP in inorganic matrix of flowers like nanomaterial (Wang et al., 2014). **(B)** Covalent attachment of HRP on NH_2_ –modified Fe_3_O_4_/SiO_2_ magnetic nanoparticles (Chang and Tang, 2014).

The water-soluble heme-containing peptides obtained by proteolytic digestion of cytochrome c are the microperoxidases (MP) often used to explore aspects of the chemistry of iron porphyrins, and as mimics for some reactions catalyzed by peroxidases (Prieto et al., 2006; Araujo et al., 2007; Marques, 2007). MP are not only model compounds but also useful molecules for applications in biosensors as electron carriers, photoreceptors, microzymes, and drugs. In a systematic study to define the minimal requirements for covalent attachment of hemes to c-type cytochromes, some artificial MPs were produced *in vivo* by exploiting the secretion and cyt c apparatuses of *E. coli* (Braun and Thöny-Meyer, 2004). MP-11, a MP with 11 aminoacids residues, assembles to boron nitride nanotubes which enhance catalysis due to a strong electron coupling between the active center of MP-11 and the nanotube showing that nanostructures often modulate peroxidases activity (Li et al., 2014b). The assembly of peroxidases into micelles (Prieto et al., 2006), reversed micelles (Das and Das, 2009; Maiti et al., 2012; Das et al., 2013), liposomes (Dotsikas and Loukas, 2012), supported bilayers (Leão-Silva et al., 2011), lipid monolayers at the air-water interface (Schmidt et al., 2008), organic, inorganic, metallic, magnetic or composite nanoparticles (Xu and Han, 2004; Kim et al., 2005; Araujo et al., 2007; Khaja et al., 2007; Silva et al., 2007; Chen et al., 2011; Klyachko et al., 2012; Li et al., 2012; Pan et al., 2013; Duan et al., 2014), hydrogels and microgels (Nakashima et al., 2009; González-Sánchez et al., 2011; Wu et al., 2012; Bruns et al., 2013), nanotubes (Li et al., 2014b), multilayers and nanostructured films (Kim et al., 2010; Pallarola et al., 2012; Cortez et al., 2013) has been explored for important applications such as immunoassays design (Marquette and Blum, 2009; Dotsikas and Loukas, 2012), drug delivery (Allen et al., 2011; Kotchey et al., 2013), cancer therapy (Ibañez et al., 2015), environment protection and bioremediation (Bansal and Kanwar, 2013; Duan et al., 2014), construction of sensors (Xu and Han, 2004; Kim et al., 2005; Chen et al., 2011; Pallarola et al., 2012; Chen and Chatterjee, 2013; Cortez et al., 2013), and energy production (Ramanavicius et al., 2005; Ramanavicius and Ramanaviciene, 2009). HRP adsorbs onto silicon wafers providing reusable films for emulsion polymerization (Naves et al., 2007) and is useful for tissue engineering applications by forming hydrogels *in situ* via crosslinking (Bae et al., 2014) and for atom transfer radical polymerization (Bruns et al., 2013). Although the enzyme-catalyzed polymerizations are environmentally advantageous, the high cost, large quantity of enzymes required for polymerization and formation of relatively low molecular weight polymers obstruct their employment in the industry (Albertsson and Srivastava, 2008). Catalase and superoxide dismutase modified by poly (ethylene glycol) (PEG) or encapsulated in PEG-coated liposomes have increased bioavailability and enhanced protection of the enzymatic activity in animal models. Pluronic-based micelles formed with antioxidant enzymes or PEG copolymers also protect catalase and superoxide dismutase from proteolysis improving delivery of these enzymes to vascular endothelial cells. Liposomes, protein conjugates and magnetic nanoparticles also successfully deliver these antioxidant enzymes to sites of vascular oxidative stress (Hood et al., 2011). Nanoparticles of polyethylene glycol and poly-lactic/poly-glycolic acid encapsulate catalase and HRP into ~300 nm diameter nanospheres combating the oxidative stress both in cell culture and in animals (Dziubla et al., 2008). Different cross-linking strategies and certain reaction conditions of pH and polycation/proteincharge ratio allow functional immobilization of superoxide dismutase (SOD1) and catalase in cross-linked nanoparticles made of cationic block copolymers such as polyethyleneimine-PEG (PEI-PEG) or poly(L-lysine)-PEG (Klyachko et al., 2012). In mice, ^125^I-labeled SOD1-containing nanoparticles display increased stability in blood and in the brain, and improved accumulation in brain tissues, in comparison with non-cross-linked complexes and native SOD1 (Klyachko et al., 2012). Catalase self-assembles in a cationic block copolymer of PEI-PEG yielding stable complexes with ca. 60–100 nm in size that retain antioxidant activity with negligible cytotoxicity (Batrakova et al., 2007). These particles are rapidly, in 40–60 min, taken up by bone-marrow-derived macrophages and retain catalytic activity for more than 24 h being released in active form whereas “naked” catalase is quickly degraded; about 0.6% of the injected dose locates in the brain (Batrakova et al., 2007). Catalytically active enzyme aggregates of HRP cross-linked by glutaraldehyde yield 83% of activity recovery when compared with the native enzyme; the advantage of this procedure is the possibility of including other stabilizing proteins in the aggregate such as albumin (Šulek et al., 2011). Eventually HRP immobilization by physical adsorption can be more effective than immobilization by crosslinking (Tatsumi et al., 1996). Figure 2 shows examples of immobilized HRP by entrapment in an inorganic, flower-like inorganic matrix (Figure 2A), or by covalent attachment to hybrid particles (Figure 2B).

A strategy receiving a wide spread interest is the use of peroxidase mimetic systems with intrinsic peroxidase-like activities associated to reliable reactivity and facile production and storage. Hemin, hematin and porphyrin (Johnstone et al., 1997; Wang et al., 2007; Griffith et al., 2012), magnetic nanoparticles and nanocomposites (Dai et al., 2009; Park et al., 2011; Gao et al., 2013), metals and alloys (Bernsmann et al., 2011; Sun et al., 2013; Zhou et al., 2013), metal oxides and sulfides (Asati et al., 2009; He et al., 2012; Hong et al., 2013), carbon materials (Shi et al., 2011; Li et al., 2013), and others (Wang et al., 2013) have been described and praised for their intrinsic peroxidase-like activities in a variety of assays. For non-aqueous applications, the peroxidase-mimetic materials are more convenient and versatile than the natural enzymes. For example, tungsten carbide (WC) as catalyst for electron-transfer reactions has been extensively investigated for applications in fuel cells and oxygen reduction (Palanker et al., 1976; Rosenbaum et al., 2006), because it exhibits catalytic properties similar to those of noble metals (Levy and Boudart, 1973). Recently, the intrinsic catalytic activity of tungsten carbide nanorods toward typical peroxidase substrates was described in comparison to the one of natural HRP leading to the demonstration that the WC NRs have excellent catalytic behavior in various organic media, are stable and reusable representing a promising peroxidase mimic (Li et al., 2014a).

Layer-by-layer deposition of anionic and cationic polyelectrolytes plus catalase—gold nanoparticles composites originates multilayers onto electrodes that effectively permit electron transfer between catalase and the electrode at high loading of the composites (Kim et al., 2010). Following a similar approach the polyelectrolyte DNA organizes an enzyme cascade of discrete glucose-oxidase and HRP pairs with controlled inter-enzyme distance resulting in enhanced enzymatic activity at short distances (Fu et al., 2012). DNA also directs HRP immobilization onto porous SiO_2_ thin films resulting in high enzymatic activity (Shtenberg et al., 2012). Peroxidases degrade carbon nanotubes *in vitro* and *in vivo* so that carbon-based nanocarriers specifically designed to target organs and cells can deliver their cargo, and biodegrade via peroxidase-driven mechanism, a really attractive therapeutic delivery option in nanomedicine (Shvedova et al., 2012; Kotchey et al., 2013). Similarly, peroxidases can also release the hydrophobic cargo of poly (propylene sulfide) nanoparticles in the presence of H_2_O_2_ (Allen et al., 2011).

Remediation of phenol waste water by immobilized HRP in magnetic nanoparticles involves the oxidation of phenols in the presence of H_2_O_2_ and the reaction of the phenoxy radicals with each other in a non-enzymatic process forming polymers; the polymers are then removed by precipitation with salts or condensation and the reusable magnetic nanoparticles with improved HRP activity are rescued (Duan et al., 2014). The importance of peroxidases for treating and removing pollutants such as phenols and halogenated phenols, polycyclic aromatic hydrocarbons (PAH), endocrine disruptive chemicals (EDC), pesticides, dioxins, polychlorinated biphenyls (PCB), industrial dyes, and other xenobiotics has been recognized and recently reviewed (Bansal and Kanwar, 2013). The recent efforts on the rational design of supramolecular hemoprotein assemblies have also been recently reviewed (Oohora and Hayashi, 2014).

For biomedical and technological applications, peroxidases in nanostructures represent a rapidly growing area for innovative research improving enzyme activity, protection, targeting, and reusability. Free peroxidases have some inherent disadvantages including loss of catalytic activity in solution during the reaction, high cost and lack of reusability. To overcome these issues, several supports including various nanomaterials for instance metal and magnetic nanomaterials, polymer based nanofibers, silicon and carbon based nanomaterials, have been used for peroxidases immobilization (Lei et al., 2002; Dulay et al., 2005; Sheldon, 2007; Wang et al., 2007; Lee et al., 2009; Cao et al., 2010; Zhu et al., 2013). In general, enhancement in enzyme activity and stability is desirable for given reactions or applications. A decrease in activity compared to the one of the free enzyme is due to the following reasons: (1) use of organic solvent for the immobilization procedure, which deactivates some parts of enzyme; (2) partially blocking of enzyme active sites during the immobilization process, which makes enzyme less accessible to the substrate; (3) unfavorable enzyme conformations on external supports; (4) mass-transfer limitations between enzymes and substrates (Luckarift et al., 2004; Kim et al., 2008; Ge et al., 2009). Nanoparticles and other nanomaterials have been the driving force behind the development of sophisticated biosensors in recent years (Bhakta et al., 2015). The use of metal nanoparticles, such as gold and platinum, results in improved rate of electron transfer at the interface. Nanofilms also detect several analytes and several are useful in the rapidly growing biosensors area. The tunable feature of nanotubes also represents a way for improving the analytical performance of biosensors. The importance of nanomaterials is growing as the demand for quick, selective, inexpensive, stable, and reproducible analytical devices continues to surge. Bioactivity and compatibility of the enzyme/material nanocomposites is a critical consideration to further the applicability of biosensors (Bhakta et al., 2015). A immobilization approach to obtain protein-inorganic hybrid nanoflowers greatly increases enzyme activity and stability (Ge et al., 2012). HRP and Fe^2+^ ions together yield flowerlike hybrid nanostructures, the hybrid nanoflowers (HNF) that enhance HRP activity by 5–7 times when compared to free HRP; this is due to high local HRP concentration, appropriate HRP conformation, less mass transfer limitations, and Fe^2+^ activation (Ocsoy et al., 2015). Therefore, peroxidases and their mimetic systems in nanostructures (like the nanoflowers) represent a flourishing field for biomedical and technological research.

### Conflict of interest statement

The authors declare that the research was conducted in the absence of any commercial or financial relationships that could be construed as a potential conflict of interest.
